# Effect of Volume on Postoperative Outcomes After Left Pancreatectomy: A Multicenter Prospective Snapshot Study (SPANDISPAN Project)

**DOI:** 10.3390/jcm14176013

**Published:** 2025-08-25

**Authors:** Daniel Aparicio-López, José M. Ramia, Celia Villodre, Juan J. Rubio-García, Belén Hernández, Juli Busquets, Luis Secanella, Nuria Peláez, Maialen Alkorta, Itziar de-Ariño-Hervás, Mar Achalandabaso, Enrique Toledo-Martínez, Fernando Rotellar, Pablo Martí-Cruchaga, Miguel A. Gómez-Bravo, Gonzalo Suárez-Artacho, Marina Garcés-Albir, Luis Sabater, Gabriel García-Plaza, Francisco J. Alcalá, Enrique Asensio, David Pacheco, Esteban Cugat, Francisco Espín, María Galófre-Recasens, Belinda Sánchez-Pérez, Julio Santoyo-Santoyo, Jorge Calvo, Carmelo Loinaz, María I. García-Domingo, Santiago Sánchez-Cabús, Belén Martín-Arnau, Gerardo Blanco-Fernández, Isabel Jaén-Torrejimeno, Carlos Domingo-del-Pozo, Carmen Payá, Carmen González, Eider Etxebarría, Rafael López-Andújar, Cristina Ballester, Ana B. Vico-Arias, Natalia Zambudio-Carroll, Sergio Estévez, Manuel Nogueira-Sixto, José I. Miota, Belén Conde, Miguel A. Suárez-Muñoz, Jorge Roldán-de-la-Rua, Angélica Blanco-Rodríguez, Manuel González, Pilar E. González-de-Chaves-Rodríguez, Betsabé Reyes-Correa, Santiago López-Ben, Berta Tió, Javier Mínguez, Inmaculada Lasa-Unzué, Alberto Miyar, Lorena Solar, Fernando Burdío, Benedetto Ielpo, Alberto Carabias, María P. Sanz-Muñoz, Alfredo Escartín, Fulthon Vela, Elia Marqués, Adelino Pérez, Gloria Palomares, Antonio Calvo-Córdoba, José T. Castell, María J. Castro, María C. Manzanares, Enrique Artigues, Juan L. Blas, Luis Díez, Alicia Calero, José Quiñones, Mario Rodríguez, Cándido F. Alcázar-López, Mario Serradilla-Martín

**Affiliations:** 1 Department of Surgery, Hospital Universitario San Jorge, 22004 Huesca, Spain; dapariciol@salud.aragon.es; 2 Department of Surgery, Hospital General Universitario Dr. Balmis, 03010 Alicante, Spain; ramia_jos@gva.es (J.M.R.); cvillodre@umh.es (C.V.);; 3 ISABIAL, 03010 Alicante, Spain; 4 Department of Surgery, School of Medicine, Miguel Hernández University, 03202 Alicante, Spain; 5 Department of Surgery, Hospital General Universitario de Elda, 03600 Alicante, Spain; 6 Department of Surgery, Hospital Universitario de Bellvitge, 08907 Barcelona, Spain; jbusquets@bellvitgehospital.cat (J.B.); lsecanella@bellvitgehospital.cat (L.S.);; 7 Department of Surgery, Hospital Universitario de Donostia, 20014 San Sebastián, Spain; maialen.alkortazuloaga@osakidetza.eus (M.A.); itziar.dearinohervas@osakidetza.eus (I.d.-A.-H.); 8 Department of Surgery, Hospital Universitario Marqués de Valdecilla, 39008 Santander, Spain; mariadelmar.achalandabaso@scsalud.es (M.A.);; 9 Department of Surgery, Clínica Universidad de Navarra, 31008 Pamplona, Spain; frotellar@unav.es (F.R.); pamartic@unav.es (P.M.-C.); 10 Department of Surgery, Hospital Universitario Virgen del Rocío, 41013 Sevilla, Spain; mangel.gomez.sspa@juntadeandalucia.es (M.A.G.-B.);; 11 Department of Surgery, Hospital Clínico Universitario, University of Valencia, Biomedical Research Institute, 46010 Valencia, Spain; garces_maralb@gva.es (M.G.-A.); luis.sabater@uv.es (L.S.); 12 Department of Surgery, Hospital Insular de Gran Canaria, 35016 Las Palmas, Spain; ggarpla@gobiernodecanarias.org (G.G.-P.); javi7alcserr@hotmail.com (F.J.A.); 13 Department of Surgery, Hospital Universitario Río Hortega, 47012 Valladolid, Spain; easensiodi@saludcastillayleon.es (E.A.); dpachecosa@saludcastillayleon.es (D.P.); 14 Department of Surgery, Hospital Universitario German Trias i Pujol, 08916 Badalona, Spain; ecugata.germanstrias@gencat.cat (E.C.); mariagalofre@uic.es (M.G.-R.); 15 Department of Surgery, Hospital Regional Universitario, 29010 Málaga, Spain; belinda.sanchez.sspa@juntadeandalucia.es (B.S.-P.); jsantoyo@telefonica.net (J.S.-S.); 16 Department of Surgery, Hospital Universitario Doce de Octubre, 28041 Madrid, Spain; jorge.calvo@salud.madrid.org (J.C.); carmelo.loinaz@salud.madrid.org (C.L.); 17 Department of Surgery, Hospital Mutua de Tarrasa, 08221 Tarrasa, Spain; maribelgarcia@mutuaterrassa.es; 18 Department of Surgery, Hospital Universitario Santa Creu i Sant Pau, 08025 Barcelona, Spain; ssanchezca@santpau.cat (S.S.-C.); amartinar@santpau.cat (B.M.-A.); 19 Department of Surgery, Hospital Universitario de Badajoz, 06080 Badajoz, Spain; gerardoblanco@unex.es (G.B.-F.); isabel.jaen@salud-juntaex.es (I.J.-T.); 20 Department of Surgery, Hospital Universitario Doctor Peset, 46017 Valencia, Spain; domingo_cardel@gva.es (C.D.-d.-P.); paya_car@gva.es (C.P.); 21 Department of Surgery, Hospital Universitario de Basurto, 48013 Bilbao, Spain; mariacarmen.gonzalezserrano@osakidetza.eus (C.G.); eider.etxebarriabeitia@osakidetza.eus (E.E.); 22 Department of Surgery, Hospital Universitario y Politécnico La Fe, 46026 Valencia, Spain; rafaellopezandujar@gmail.com (R.L.-A.); cris7balle@yahoo.es (C.B.); 23 Department of Surgery, Hospital Universitario Virgen de las Nieves, 18014 Granada, Spain; anab.vico.sspa@juntadeandalucia.es (A.B.V.-A.); natalia.zambudio.sspa@juntadeandalucia.es (N.Z.-C.); mserradilla@ugr.es (M.S.-M.); 24 Department of Surgery, Complejo Hospitalario Universitario de Vigo, 36312 Vigo, Spain; sergio.manuel.estevez.fernandez@sergas.es (S.E.); manuel.nogueira.sixto@sergas.es (M.N.-S.); 25 Department of Surgery, Hospital General Universitario de Albacete, 02008 Albacete, Spain; jimiotad@sescam.jccm.es (J.I.M.); bciconde@gmail.com (B.C.); 26 Department of Surgery, Hospital Universitario Virgen de la Victoria, 29010 Málaga, Spain; mangel.suarez.sspa@juntadeandalucia.es (M.A.S.-M.);; 27 Department of Surgery, Hospital Universitario de A Coruña, 15006 A Coruña, Spain; 28 Department of Surgery, Hospital Universitario Nuestra Señora de la Candelaria, 38010 Tenerife, Spain; pancreashunsc@gmail.com (P.E.G.-d.-C.-R.); bereyes86@hotmail.com (B.R.-C.); 29 Department of Surgery, Hospital Universitario Dr. Josep Trueta, 17007 Girona, Spain; slopezben.girona.ics@gencat.cat (S.L.-B.); btiomuntadas@gmail.com (B.T.); 30 Department of Surgery, Hospital Universitario Príncipe de Asturias, 28805 Alcalá de Henares, Spain; javier.minguez@salud.madrid.org (J.M.); inmaculada.lasa@uah.es (I.L.-U.); 31 Department of Surgery, Hospital Universitario Central de Asturias, 33011 Oviedo, Spain; alberto.miyarde@sespa.es (A.M.); lorena.solar@sespa.es (L.S.); 32 Department of Surgery, Hospital del Mar, 08003 Barcelona, Spain; fburdio@hotmail.com (F.B.); bielpo@hmar.cat (B.I.); 33 Department of Surgery, Hospital Universitario de Getafe, 28905 Getafe, Spain; carabher@yahoo.es (A.C.);; 34 Department of Surgery, Hospital Universitario Arnau de Vilanova, 25198 Lleida, Spain; aescartin.lleida.ics@gencat.cat (A.E.); ffvela.lleida.ics@gencat.cat (F.V.); 35 Department of Surgery, Hospital Universitario Infanta Leonor, 28031 Madrid, Spain; elia.marques@salud.madrid.org (E.M.); apperezm@salud.madrid.org (A.P.); 36 Department of Surgery, Hospital Universitario Morales Meseguer, 30008 Murcia, Spainantonio.calvo@carm.es (A.C.-C.); 37 Department of Surgery, Hospital Universitario La Luz, 28003 Madrid, Spain; jtcastell@quironsalud.es; 38 Department of Surgery, Hospital Universitario Puerta del Mar, 11009 Cádiz, Spain; 39 Department of Surgery, Hospital General Universitario de Ciudad Real, 13005 Ciudad Real, Spain; mcmc09@sescam.jccm.es; 40 Department of Surgery, Hospital General de Valencia, 46014 Valencia, Spain; artigues_enr@gva.es; 41 Department of Surgery, Hospital Royo Villanova, 50015 Zaragoza, Spain; jlblas@salud.aragon.es; 42 Department of Surgery, Hospital Clínico de San Carlos, 28040 Madrid, Spain; lidiez@hotmail.com; 43 Department of Surgery, Hospital Universitario de Elche, 03203 Elche, Spain; calero_ali@gva.es; 44 Department of Surgery, Hospital Universitario Salamanca, 37007 Salamanca, Spain; jequinones@saludcastillayleon.es; 45 Department of Surgery, Hospital Clínico de Valladolid, 47007 Valladolid, Spain; mariorodriguezlopez@gmail.com; 46 Instituto de Investigación Biosanitaria ibs.GRANADA, School of Medicine, University of Granada, 18016 Granada, Spain

**Keywords:** left pancreatectomy, volume, outcomes, surgery, regionalization

## Abstract

**Background/Objectives:** Like many other countries, the management of pancreatic cancer in Spain has developed in a fragmented manner. This study analyzes clinical outcomes related to patient volume at different centers after left pancreatectomy (LP). Our goal is to determine whether our practices align with the standards established in the literature and assess whether centralization’s advantages significantly outweigh its disadvantages. **Methods:** The SPANDISPAN Project (SPANish DIStal PANcreatectomy) is an observational, prospective, multicenter study focused on LP conducted in Spanish Hepato-Pancreato-Biliary (HPB) Surgery Units from 1 February 2022 to 31 January 2023. HPB units were defined as high volume if they performed more than 10 LPs annually. **Results:** This study included 313 patients who underwent LP at 42 centers across Spain over the course of a year. A total of 40.3% of the procedures were performed in high-volume centers. Significant differences in preoperative variables were only observed in ASA scores, which were higher in the high-volume group. Intraoperatively, minimally invasive surgical techniques were performed more frequently in high-volume centers. Postoperatively, the administration of somatostatin, major complications, and B and C postoperative pancreatic fistula (POPF) were more frequent in low-volume hospitals. **Conclusions:** The findings revealed that high-volume centers had a higher rate of minimally invasive surgery, lower intraoperative bleeding, fewer complications, and reduced POPFs compared to low-volume centers. However, it is important to note that low-volume centers still demonstrated acceptable outcomes. Thus, the selective referral of more complex laparoscopic procedures could initiate a gradual centralization of surgical practices.

## 1. Introduction

Pancreatic cancer remains a highly fatal disease with a 5-year survival rate of less than 10% despite improvements in diagnosis, surgical techniques, and systemic treatments [1]. Likewise, most patients present with locally advanced (30–35%) or metastatic (50–55%) disease at the time of diagnosis [1]. Left pancreatectomy (LP), with or without splenectomy, is the surgical technique used to treat tumors in the body and tail of the pancreas [2]. LP is associated with low mortality (<3%) but high morbidity (>30%), usually related to postoperative pancreatic fistula (POPF) [3]. Implementing the LP by minimally invasive surgery (MIS) (laparoscopic or robotic) has represented a great advance and is considered today’s approach of choice. Its main advantages are less bleeding, less need for transfusion, shorter hospital stay, rapid functional recovery, and better postoperative quality of life with the same oncological outcomes and mortality and POPF rates [3,4,5,6,7].

Improving the quality of care is an absolute priority for health systems to provide better care and reduce costs, always maintaining efficiency and guaranteeing clinical results with high-quality standards [8,9,10]. One of the alternatives to achieve these objectives is centralized resection of pancreatic cancer, which highlights its complexity, risks, and need for experience and resources [11,12,13].

Centralization policies are usually based on patient thresholds [14]. Multiple factors can affect clinical results: early diagnosis programs, multidisciplinary teams including advanced endoscopic procedures and interventional radiology, standardized protocols, updated systemic treatments, and surgical interventions with low rates of failure to rescue [11].

Centralization has several benefits, some directly related to improving clinical results (higher resection rates, fewer complications, and better survival) [15,16]. However, beyond these, they include professional development, specialized training, participation in clinical trials, safety improvement, and efficiency in introducing advances in care [17].

Worldwide centralized care for pancreatic cancer is variable because implementation rates are very heterogeneous, with very few countries able to carry it out satisfactorily. [14]. The main barriers to centralization are the geographic distance, population density, available resources, experience of health personnel, and resistance to change in practices or national health service delivery models [13].

From the rejection and concern of low-volume centers to see their volume of patients reduced, as well as budget allocation and experience, the term regionalization arises as opposed to centralization, since it better reflects the accumulation of cases in centers with higher volume to guarantee adequate support to the hospitals in an area [11].

A wealth of scientific literature analyzes the clinical outcomes of pancreatic surgery based on patient volume. Like many other countries, the management of pancreatic cancer in Spain has developed in a fragmented manner, without a coordinated effort to centralize care thus far. This study analyzes clinical outcomes related to patient volume at different centers after LP. Our goal is to determine whether our practices align with the standards established in the literature and assess whether centralization’s advantages significantly outweigh its disadvantages.

## 2. Materials and Methods

The SPANDISPAN Project (SPANish DIStal PANcreatectomy) is an observational, prospective, multicenter study focused on LP conducted in Spanish Hepato-Pancreato-Biliary (HPB) Surgery Units over one year, from 1 February 2022 to 31 January 2023. Seventy hospitals previously participating in the Spanish Association of Surgery/International Hepato-Pancreato-Biliary Association (AEC/IHPBA) Pancreatic Surgery Survey were contacted via email [18]. A centralization model is employed in one region of Spain; any hospital may perform LP in the other sixteen regions.

Each participating center assigned a local administrator to oversee data collection and liaison with the overall study coordinator. Local administrators gathered data at their respective hospitals, and a REDCap^®^ database (Research Electronic Data Capture, Vanderbilt University, Nashville, TN, USA) was established for the study.

The study adhered to the Declaration of Helsinki (2013) and was approved by the Clinical Research Ethics Committee of the Hospital General Universitario Dr. Balmis (Alicante, Spain) on 28th April 2021 (CEIm: Acta 2021-04). Patients provided informed consent before participating in the study, which is reported according to the STROBE guidelines [19].

The study included any scheduled LP performed during the study period, regardless of diagnosis, in patients over 18 years of age. Exclusion criteria included LP with celiac trunk resection, LP after pancreaticoduodenectomy, or emergency LP. The suspected preoperative diagnosis was based on imaging tests such as CT, MRI, and EUS. The surgical approach utilized could be open or minimally invasive (laparoscopic or robotic), with or without spleen preservation.

### 2.1. Variables and Definitions

The variables studied include demographic data such as age, sex, body mass index (BMI), history of previous abdominal surgeries, medications, and the ASA (American Society of Anesthesiologists) scale score [20]. Additional data were collected on biological symptoms, radiological findings, and the surgical approach utilized (open, laparoscopic, or robotic). Conversion is defined as a change from minimally invasive surgery (MIS) to open laparotomy. Spleen preservation refers to using the Warshaw or Kimura techniques, while associated organ resection is the removal of at least one additional organ, excluding the spleen. Intraoperative blood loss and the need for transfusion were also recorded.

Postoperative data included morbidity and mortality, with complications evaluated at 90 days using the Clavien–Dindo classification system [21]. Complications classified as grade IIIa or higher were considered major. The complications were documented based on medical and nursing clinical notes from each patient’s electronic medical records. Specific definitions applied to pancreatic surgery complications were drawn from the International Study Group on Pancreatic Surgery (ISGPS) guidelines for delayed gastric emptying (DGE) [22], post-pancreatectomy hemorrhage (PPH) [23], and POPF [24].

The resection margins of the specimens were classified according to the Royal College of Pathologists’ definitions: R0 (tumor margin ≥ 1 mm), R1 (tumor margin < 1 mm), and R2 (macroscopically positive margin) [25]. We classified invasive tumors using the TNM classification system (8th edition) [26]. Reintervention was defined as any unscheduled surgical procedure related to pancreatic resection. Hospital stays and readmissions were measured within 90 days. The histological data collected included tumor size, R status, and the size of the resected pancreas.

We used a reference of 10 patients to categorize participating centers into low and high volumes [27]. Centers that reported 10 or fewer patients during the study period were classified as low volume, while those that reported more than 10 patients were categorized as high volume.

### 2.2. Statistical Analysis

Measurements were conducted using Microsoft^®^ Excel for Mac, version 16.49, and SPSS^®^ for Mac, version 26.0 (SPSS Inc., Chicago, IL, USA). All calculations were performed with R (version 4.2.1).

Descriptive statistics were computed using frequencies and percentages for categorical data and medians and interquartile ranges (IQR) for continuous data. The IQR represents the range between the first quartile (Q1) and the third quartile (Q3). Categorical variables were described by indicating the number of cases and their respective percentages. The chi-square test was employed to assess the association between two categorical variables. The relevant data were collected and organized in a contingency table, after which the chi-square test with Yates’ correction was applied. The Mann–Whitney U test was utilized to compare the distributions of two nonparametric continuous variables.

A logistic regression model was implemented to analyze the association between predictor variables and a binary outcome variable, with odds ratios calculated to measure the strength of the association. Point estimates of the odds ratios and their corresponding 95% confidence intervals (95% CI) were generated.

## 3. Results

**Figure 1:**
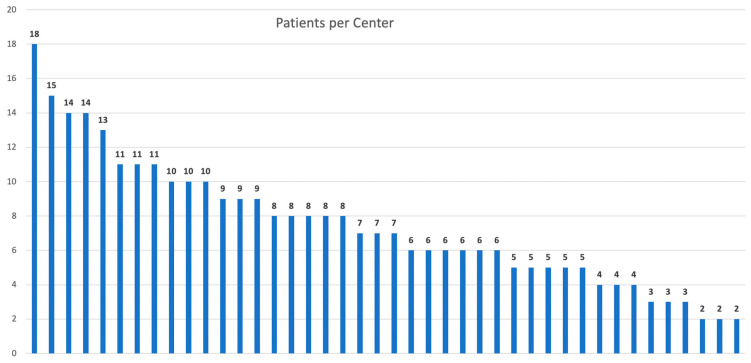
Patients operated on at each participating center in Spain.

This study included 313 patients who underwent LP at 42 centers across Spain over the course of a year. A total of 40.3% of the procedures were performed in high-volume centers (>10 cases/year). The median number of LPs conducted per center was 7, with an interquartile range (IQR) of 5 to 10 (Figure 1).

The median age of the patients was 65 years (IQR 55–74), and 53.4% were women. The mean body mass index (BMI) was 27.4 kg/m^2^ (IQR 24.0–30.5). The most common ASA score among the patients was II, representing 47.6%, and the median Charlson Comorbidity Index was 4 (IQR 2–5) (Table 1).

In terms of tumor location, 40.6% of cases involved the tail of the pancreas. The primary indications for surgery were neuroendocrine tumors, accounting for 31.0% of cases, and pancreatic adenocarcinoma, which comprised 26.2%. The median tumor size was 28 mm (IQR 17–44) (Table 2).

In 69.3% of the patients, MIS was used, and the most frequently performed procedure was LP with splenectomy, which occurred in 86.6% of the cases. Preservation of the spleen was achieved in 13.4% of patients. The median operative time was 240 min (IQR 180–300) (Table 2). Major complications were observed in 23.7% of patients, with postoperative pancreatic fistula (POPF) grades B and C occurring in 20.1% of cases. The 90-day mortality rate was 1.6%. R0 resection was achieved in 92% of the LPs.

A total of 187 patients were included in the low-volume group from 34 centers (59.7%), while 126 were in the high-volume group from 8 centers (40.3%). Significant differences in preoperative variables were only observed in ASA scores, which were higher in the high-volume group. The distribution by type of tumor and tumoral size did not show statistical differences (Table 1).

Intraoperatively, minimally invasive surgical techniques, including laparoscopic and robotic approaches, were performed more frequently in high-volume centers. However, the conversion rate was also higher in this group. Using a 60 mm stapler and omental patch following LP was more common in high-volume centers, where intraoperative blood loss was lower (Table 2).

Postoperatively, the administration of somatostatin was more prevalent in low-volume hospitals. Major complications, assessed according to the Clavien–Dindo classification, were also more frequent in low-volume hospitals. Clinically relevant POPF (CR-POPF) was higher in low-volume hospitals. DGE, PPH, and non-pancreas-related complications were similar in both groups.

No differences were observed in the length of stay or readmission rates between the two groups. There were no differences concerning tumor type, margin status, or the number of lymph nodes harvested (Table 3).

## 4. Discussion

In our prospective study involving 313 LPs, 60% were conducted in low-volume centers. Centers that perform more than 10 LPs per year demonstrated a higher percentage of minimally invasive surgeries, experienced fewer complications and CR-POPF, and employed certain technical and management variations. These included using larger staplers, a more frequent application of the omental patch, and a reduced reliance on somatostatin.

Central pancreatic surgery has multiple proven benefits [13,14,15,16,17,28,29,30]. Still, manuscripts about this topic usually focus on patients undergoing pancreaticoduodenectomy, as LP is generally regarded as less complex, with lower mortality and morbidity rates [31]. However, LP is not without complications; for instance, POPF can occur in up to 30% of patients. This complication may lead to more extended hospital stays, increased reoperations, and delays in initiating adjuvant treatment for patients with malignant tumors [32]. Therefore, centralizing LP could yield advantages like those in more complex surgeries. Successful outcomes are not solely dependent on the surgical technique; having multidisciplinary teams that include 24/7/365 endoscopists, interventional radiologists, and other specialists is crucial for improving patient outcomes [28].

When we proposed this study, our main question was whether low-volume centers might select fewer complex patients, which could lead to a biased comparison between the two groups. The only difference we observed was that patients from high-volume centers had a higher ASA score. This suggests their greater experience, and a more expert multidisciplinary team may enable them to treat more fragile patients. We did not find any other patient or tumor characteristics that distinguished the two groups, which allows for a valid comparison. In a previous benchmarking analysis of our series, we found that low-volume centers also did not select simpler patients, as the number of patients in the low-risk group (BMI < 35 kg/m^2^, ASA < III, no multivisceral resection, no previous liver and/or pancreas surgery, use of any surgical approach, and any diagnosis) was similar between low- and high-volume centers [33].

Previous studies have defined several cut-offs, 15 LP for example, for defining high- or low-volume centers [34,35]. Even more, three groups of low, intermediate, and high-volume hospitals have been used before. We used a 10 LP cut-off due to the distribution of the number of cases per center [27] (Figure 1).

We want to highlight technical and management aspects that differentiate the two groups. Currently, minimally invasive surgery is recognized as the gold standard for LP, achieving a total rate of 70% that improves the latest published series [27]. It is logical that higher-volume centers perform more minimally invasive surgeries and are the first to adopt robotic surgery, which will likely become the preferred technique [36]. An omental patch after LP is a technical trick that is being performed more frequently. Higher-volume groups tend to use this technique more regularly, likely due to their greater experience in pancreatic surgery [37].

We have no explanation for the differences observed in the use of various sizes of staplers, as there are no significant differences between the two groups concerning the cut area or pancreatic thickness. Furthermore, somatostatin has not demonstrated a decrease in POPF rates and is, therefore, not currently recommended for prophylactic use [36,37,38,39,40]. We hypothesize that the higher rate of POPF in low-volume hospitals may explain the increased use of somatostatin in those settings.

This study’s limitation lies in its multicenter design, which includes various surgical teams, each following its own protocols. This variability could introduce inconsistencies in data collection and analysis. Due to the short follow-up, we do not know if oncological results are also linked to the center’s volume. However, a notable strength of the study is its prospective nature, which compares low-volume centers with high-volume centers. Recent data collection shows a high rate (70%) of MIS.

## 5. Conclusions

In conclusion, 60% of patients underwent surgery in low-volume centers. The findings revealed that high-volume centers had a higher rate of MIS, along with specific technical variations (such as omental patch techniques and stapler lengths), lower intraoperative bleeding, fewer complications, and reduced POPFs compared to low-volume centers. However, it is important to note that low-volume centers still demonstrated acceptable outcomes. Thus, the selective referral of more complex laparoscopic procedures could initiate a gradual centralization of surgical practices.

## Figures and Tables

**Table 1:** Comparison of preoperative variables between low and high-volume centers.

	Total *N = 131*	Low-Volume (n ≤ 10) *N = 187*	High-Volume (n > 10) *N = 126*	* p * Value
** Age, years (IQR) **	65.0 [55.0;74.0]	64.0 [54.5;73.0]	67.0 [57.2;75.0]	0.227
** Gender, n (%) **				0.389
**Male**	146 (46.6)	83 (44.4)	63 (50.0)	
**Female**	167 (53.4)	104 (55.6)	63 (50.0)	
** Comorbidity Charlson Index, median (IQR) **	4.00 [2.00;5.00]	3.00 [2.00;5.00]	4.00 [2.00;5.00]	0.800
** Body Mass Index, kg/m^2^ (IQR) **	27.4 [24.0;30.5]	27.0 [23.8;30.1]	27.8 [24.2;31.0]	0.340
** ASA score, n (%) **				0.038
**I**	17 (5.4)	13 (6.9)	4 (3.2)	
**II**	149 (47.6)	92 (49.2)	57 (45.2)	
**III**	141 (45.0)	76 (40.6)	65 (51.6)	
**IV**	6 (1.9)	6 (3.2)	0 (0.0)	

IQR: interquartile range; ASA: American Society of Anesthesiologists.

**Table 2:** Comparison of intraoperative variables between low and high-volume centers.

	Total *N = 313*	Low-Volume (n ≤ 10) *N = 187*	High-Volume (n > 10) *N = 126*	* p * Value
** Tumor location, n (%) **				0.203
** Tail **	127 (40.6)	71 (38.0)	56 (44.4)	
** Body **	86 (27.5)	49 (26.2)	37 (29.4)	
** Body-tail **	77 (24.6)	54 (28.9)	23 (18.3)	
** Neck **	23 (7.4)	13 (7.0)	10 (7.9)	
** Histology, n (%) **				
** NET **	97 (31.0)	55 (29.4)	42 (33.3)	0.723
** Adenocarcinoma **	82 (26.2)	50 (26.7)	32 (25.4)	0.660
** IPMN **	33 (10.5)	18 (9.6)	15 (11.9)	0.231
** Mucinous cystic neoplasm **	26 (8.3)	13 (7.0)	13 (10.3)	0.814
** Serous cystadenoma **	19 (6.1)	12 (6.4)	7 (5.6)	1.000
** Pancreatic metastasis **	9 (2.9)	5 (2.7)	4 (3.2)	0.718
** Pancreatic pseudocyst **	7 (2.2)	7 (3.7)	0 (0.0)	0.517
** Solid pseudopapillary tumor **	4 (1.3)	2 (1.1)	2 (1.6)	0.225
** Other **	36 (11.5)	25 (13.4)	11 (8.7)	0.167
** Tumor size, median (IQR) **	28.0 [17.0;44.0]	29.0 [18.0;45.0]	27.0 [15.5;38.0]	0.358
** Surgical technique, n (%) **				0.907
** Left pancreatectomy **	226 (72.2)	136 (72.7)	90 (71.4)	
** RAMPS **	45 (14.4)	25 (13.4)	20 (15.9)	
** Spleen-preserving pancreatectomy **	42 (13.4)	26 (13.9)	16 (12.7)	
** Approach, n (%) **				0.046
** Laparoscopic **	166 (53.0)	94 (50.3)	72 (57.2)	
** Robotic **	51 (16.3)	26 (13.9)	25 (19.8)	
** Open **	96 (30.7)	67 (35.8)	29 (23.0)	
** Conversion, n (%) **	23 (10.6%)	8 (6.7%)	15 (15.5%)	0.061
** Pancreas consistency, n (%) **				0.668
** Soft **	183 (58.5)	107 (57.2)	76 (60.3)	
** Hard **	130 (41.5)	80 (42.8)	50 (39.7)	
** Stapler for closing pancreatic stump, n (%) **	278 (88.8)	168 (89.8)	110 (87.3)	0.606
** Length of stapler, n (%) **				0.006
** 45 mm **	15 (5.86)	14 (9.0)	1 (1.0)	
** 60 mm **	234 (91.4)	135 (87.1)	99 (98.0)	
** Other **	7 (2.7)	6 (3.9)	1 (1.0)	
** Use of epiploplasty, n (%) **	17 (5.4)	3 (1.6)	14 (11.1)	0.001
** Intraoperative loss of blood, ml (IQR) **	120 [50.0;300]	150 [100;300]	100 [0.00;288]	0.003
** Intraoperative transfusion, n (%) **	20 (6.4)	10 (5.4)	10 (7.9)	0.495
** Other organs resected (not including spleen), n (%) **	90 (28.8)	73 (39.0)	17 (13.5)	0.768
** Operative time, min (IQR) **	240 [180;300]	240 [180;284]	240 [190;300]	0.367
** Use of abdominal drain (yes), n (%) **	282 (90.1)	173 (92.5%)	109 (86.5%)	0.121

NET: neuroendocrine tumor; IPMN: intraductal papillary mucinous neoplasiaRAMPS: radical antegrade modular pancreatosplenectomy; IQR: interquartile range.

**Table 3:** Comparison of postoperative variables between low and high-volume centers.

	Total *N = 313*	Low-Volume (n ≤ 10) * N = 187 *	High-Volume (n > 10) *N = 126*	* p * Value
** Postoperative complications, n (%) **				
** No **	180 (57.5)	107 (57.2)	73 (57.9)	0.993
** Clavien–Dindo I **	86 (27.5)	40 (21.4)	46 (36.5)	0.005
** Clavien–Dindo II **	59 (18.8)	36 (19.3)	23 (18.3)	0.941
** Clavien–Dindo IIIa **	48 (15.3)	36 (19.3)	12 (9.5)	0.029
** Clavien–Dindo IIIb **	14 (4.5)	8 (4.3)	6 (4.8)	1.000
** Clavien–Dindo IVa **	8 (2.6)	6 (3.2)	2 (1.6)	0.482
** Clavien–Dindo IVb **	1 (0.3)	0 (0.0)	1 (0.8)	0.403
** Clavien–Dindo V **	3 (1.0)	2 (1.1)	1 (0.8)	1.000
** Clavien–Dindo ≥ IIIa **	74 (23.7)	52 (27.9)	22 (17.5)	0.023
** Comprehensive Complication Index, median (IQR) **	8.70 [0.00;20.9]	8.70 [0.00;26.2]	8.70 [0.00;20.9]	0.242
** POPF, n (%) **				0.255
** Biochemical **	57 (18.2)	31 (16.6)	26 (20.6)	
** B **	53 (16.9)	38 (20.3)	15 (11.9)	
** C **	10 (3.1)	8 (4.3)	2 (1.6)	
** POPF B + C, n (%) **	63 (20.1)	46 (24.6)	17 (13.5)	0.024
** POPF, days (IQR) **	16.0 [9.00;30.0]	20.0 [10.8;30.0]	15.0 [8.00;26.0]	0.074
** Delayed gastric emptying, n (%) **	9 (2.9)	6 (3.2)	3 (2.4)	0.745
** Postoperative hemorrhage, n (%) **	17 (5.4)	8 (4.3)	9 (7.1)	0.400
** Use of somatostatine, n (%) **	99 (31.6)	42 (22.5)	57 (45.2)	<0.001
** Reintervention, n (%) **	25 (8.0)	18 (9.6)	7 (5.6)	0.276
** Interventional radiology **	5 (1.6)	5 (2.7)	0 (0.0)	0.085
** Endoscopic **	5 (1.6)	4 (2.1)	1 (0.8)	0.652
** Surgical **	22 (7.0)	15 (8.0)	7 (5.6)	0.541
** Non-pancreas-related complications, n (%) **	50 (16.0)	31 (16.6)	19 (15.1)	0.843
** Length of stay, days (IQR) **	7.00 [5.00;9.00]	7.00 [5.00;9.50]	7.00 [5.00;9.00]	0.645
** Readmission, n (%) **	69 (22.0)	45 (24.1)	24 (19.0)	0.362
** 90-day mortality, n (%) **	5 (1.6)	2 (1.1)	3 (2.4)	0.397
** Margin status, n (%) **				0.566
** R0 **	288 (92.0)	172 (92.0)	116 (92.1)	
** R1 **	24 (7.7)	15 (8.0)	9 (7.1)	
** R2 **	1 (0.3)	0 (0.0)	1 (0.8)	
** Lymph nodes harvested, median (IQR) **	8.00 [3.00;15.00]	7.00 [3.50;15.0]	8.00 [2.25;16.0]	0.843
** Postoperative Diabetes Mellitus, n (%) **				0.098
** No **	220 (70.3)	123 (65.8)	97 (77.0)	
** Worsening **	52 (16.6)	35 (18.7)	17 (13.5)	
** New **	41 (13.1)	29 (15.5)	12 (9.5)	
**Pancreatic exocrine insufficiency, n (%)**	68 (21.7)	41 (21.9)	27 (21.4)	1.000

IQR: interquartile range; POPF: postoperative pancreatic fistula.

## Data Availability

The datasets used and/or analyzed during the current study are available from the corresponding author on reasonable request.

## Abbreviations

The following abbreviations are used in this manuscript:

AEC/IHPBA	Spanish Association of Surgery/International Hepato-Pancreato-Biliary Association
SPANDISPAN	SPANish DIStal PANcreatectomy
ISGPS	International Study Group on Pancreatic Surgery
POPF	Postoperative pancreatic fistula
MIS	Minimally invasive surgery
HPB	Hepato-Pancrea-Biliary
BMI	Body Mass Index
DGE	Delayed gastric emptying
PPH	Post-pancreatectomy hemorrhage
IQR	Interquartile range
LP	Left pancreatectomy
